# Vitamin D levels among children, adolescents, adults, and elders in Pakistani population: a cross-sectional study

**DOI:** 10.1186/s12889-022-14526-6

**Published:** 2022-11-08

**Authors:** Saba Arshad, Syed Jaffar Abbas Zaidi

**Affiliations:** 1 Department of Oral Biology, Dr. Ishrat-Ul-Ebad Khan Institute of Oral Health Sciences (DIKIOHS), Dow University of Health Sciences, Karachi, Pakistan; 2 Department of Oral Biology, Dow Dental College, Dow University of Health Sciences, Karachi, Pakistan

**Keywords:** 25 hydroxyvitamin D, Serum vitamin D, Vitamin D deficiency, Prevalence, Children, Adolescents, Adults

## Abstract

**Background:**

Vitamin D is not only an essential part of a healthy diet but it is also known as the sunshine hormone. It helps to absorb calcium and phosphate within the body and is essential for the development of teeth and bones in growing children. Deficiency in vitamin D causes weak bones, osteoporosis in older individuals, and osteomalacia in children. It also causes loss of alveolar bone around the teeth, increased dental cavities, and other problems associated with gum disease. It can cause depression, fatigue, and appetite loss. This study aims to observe vitamin D deficiency, insufficiency, and sufficiency among children, adults, adolescents, and elders in the Pakistani population.

**Methods:**

A cross-sectional survey was conducted with 27,880 individuals referred from the general out-patient-department (OPD) to Dow Diagnostic Research and Reference Laboratory (DDRRL) units at Dow University of Health Sciences (DUHS) Karachi, Pakistan, for a period of three months from January to March 2017. They were examined for laboratory findings of serum 25 hydroxyvitamin D levels to determine vitamin D deficiency, insufficiency, and sufficiency among all age groups of both male and female genders.

**Results:**

A total of 26,750 individuals with a mean age of 38 ± 18 years were statistically examined for laboratory findings of serum vitamin D levels. Vitamin D deficiency was observed in 56% of individuals with a mean log of 1.01 ± 0.18 ng/ml serum vitamin D levels, vitamin D insufficiency in 20% of individuals with a mean log of 1.38 ± 0.05 ng/ml serum vitamin D levels and vitamin D sufficiency in 24% individuals with a mean log of 1.63 ± 0.12 ng/ml serum vitamin D levels. The highest percentage of individuals deficient in vitamin D were children and adolescents of age ranging between 6 to 18 years.

**Conclusion:**

The findings of vitamin D deficiency in children and adolescents direct higher authorities in the public health sector to take immediate steps to screen, intervene and educate high-risk populations incorporating vitamin D supplements to establish preventive and therapeutic measures.

## Background

A classical hormone, vitamin D, is a subject of widespread research. It is associated with multiple genes carrying vitamin D receptors in the human body [1]. Vitamin D3 is the form of 1,25 dihydroxy vitamin D metabolites conjugated to vitamin D receptors, and retinoid X receptors mediate proteins responsible for signaling calcium and phosphate ions absorption. Therefore, calcium phosphate complexes maintain the skeletal and calcium balances in the human body [2]. A similar physiochemical phenomenon stimulates amelogenin proteins to differentiate the developing hard tissues of the teeth [3]. Previous literature revealed osseous and extra-osseous benefits associated with skeletal and maxillofacial tissues by intake of vitamin D supplements [4, 5].

Vitamin D also has immunological antimicrobial effects in the oral cavity by stimulating cathelicidin and other defensins [6]. Vitamin D deficiency can be associated with acute and chronic systemic diseases [7], exposure to ultraviolet B sunlight for less than 30 min per week [8], and nutritional deficiencies [9]. Vitamin D deficiency can cause rickets, muscle ache, and osteomalacia in children and adolescents [10]. It can result in molar incisor hypo-mineralization [11], with an increased risk of dental caries [12]. Maternal vitamin D deficiency can be associated with dental caries in the primary dentition [13]. The combination therapy of calcium and vitamin D supplements can improve periodontal health [14].

The nutritional status of vitamin D is determined by measuring the specific values from a laboratory analysis of blood serum. Serum vitamin D levels greater than 30 ng/ml are considered vitamin D sufficiency, less than 20 ng/ml are defined as vitamin D deficiency, and 20 to 29.9 ng/ml are defined as vitamin D insufficiency [15]. The treatment recommendation for vitamin D deficiency is about 5000 IU of vitamin D3/day for recovery and ≥ 2000 IU/day as a maintenance dose [16]. For children, 10 to 50 µg dose of vitamin D supplements per day is considered safe [10].

Vitamin D deficiency is highly prevalent worldwide [17]. Approximately 1,000 million vitamin D deficient or insufficient individuals were reported worldwide in 2007 [18], and up to 70% were observed in developing countries [19]. Females in the Middle East were found to be the most affected worldwide [20]. About 30–90% of the cases were reported among Chinese, Mongolians, Africans, and Middle Easterners [21] and 69–82% among South Asians [22]. In Pakistan, vitamin D deficiency was reported in approximately 73% of the population [8].

According to the Pakistan National Nutrition Survey 2011, the prevalence of vitamin D deficiency was 70% in pregnant women and 66% in non-pregnant women [23]. This survey provided no information on the overall status of vitamin D in Pakistan. However, in another study, 84% of asymptomatic adults in Karachi were deficient in vitamin D [24]. To prevent and treat vitamin D deficiency in Pakistan, it is important to monitor the current burden of serum vitamin D levels in the population so that health authorities can take appropriate preventative and therapeutic measures.

Several most common causes of illness and death were attributed to vitamin D deficiency in the previous observational and Mendelian randomization studies from Pakistan and worldwide as discussed in Table 1.

**Table 1:** The most common causes of illness and death attributed to vitamin D deficiency

Serum Vitamin D levels (ng/ml)	Common causes of illness and death	Risk of diseases with low serum 25 hydroxyvitamin D	Evidence from observational and Mendelian randomization studies
< 20 ng/ml	Myocardial infection (MI)	No significant association found	Barbarawi et al. 2019 [25]
	CVD mortality		
	all-cause mortality		
	CVD	Increased risk of CVD	Zhou, Selvanayagam et al. 2022 [26]
	Stroke	The higher risk among both white and black racial individuals	Judd, Morgan et al. 2016 [27]
> 30 ng/ml	MI and mortality	Lower risk	Acharya, Dalia et al. 2021 [28]
> 30 ng/ml	COVID-19 outcomes and mortality	Lower risk	Oristrell et al. 2022 [29]
> 40 ng/ml	Preterm birth	Lower risk	McDonnell, Baggerly, et al. 2017 [30]
> 40 ng/ml	Type 2 Diabetes	Lower risk of diabetes in prediabetic individuals	Davidson 2021 [31], Shah, Sameen, et al. 2021 [32]
< 30 ng/ml	Colorectal cancer	higher risk	Kareem and Majid 2022 [33]
< 30 ng/ml	Breast cancer	No significant association Higher risk	Shamsi, Afzal et al. 2021 [34] Tahir, Madiha et al. 2022 [35]
< 30 ng/ml	COVID-19	More invasive interventions, complications, and mortality rates observed	Asghar, Yasmin et al. 2022 [36]
> 30 ng/ml	Helicobacter Pylori infection	More efficient treatment response	Magsi, Kumar et al. 2021 [37]
< 30 ng/ml	Preeclampsia	Higher risk	Shahid, Ladak, et al. 2020 [38]

Children and adolescents have been observed to be the most affected group of the population with hypovitaminosis D than adults and elderly individuals in the South Asia region [39, 40]. However, limited literature was found with a sufficiently large sample of the study population to generalize the study results for all age groups and genders of the general population.

The rationale of the current study is to assess the status of vitamin D deficiency, insufficiency, and sufficiency among different age groups and genders by observing serum vitamin D levels in the population of Karachi, Sindh, Pakistan, and to find out the population at high risk of vitamin D deficiency and insufficiency among children, adolescents, adults, and elders.

The study’s primary objective is to measure the prevalence of vitamin D deficiency, insufficiency, and sufficiency among both male and female children, adolescents, adults, and elders. Therefore, the hypothesis of this study states that there is no statistically significant difference in serum vitamin D levels in males and females of all ages. This hypothesis was rejected at a *p*-value ≤ 0.05.

### Methodology

A retrospective cross-sectional study was conducted in the Department of Oral Biology, Dow University of Health Sciences (DUHS), Karachi, Sindh, Pakistan, from January 2017 to June 2017, following the STROBE statement of cross-sectional studies. The study included the secondary dataset of ambulatory individuals who came to the Dow Diagnostic Research & Reference Laboratory (DDRRL), DUHS, randomly to evaluate serum vitamin D levels on referral from a general out-patient-department (OPD).

Written informed consent was obtained from all individuals to use their anonymous information and laboratory results for serum vitamin D levels for research purposes while collecting serum samples at DDRL. Parents or legal guardians signed informed consent for the participating children and adolescents. Ethical approval was obtained from the Institutional Review Board (IRB) of DUHS with reference no. IRB-784/DUHS/Approval/2016/341 along with approval from the Director of the DDRL on 06 December 2016.

DDRRL is a rapidly expanding laboratory in which about 25,000 tests are performed monthly. There are 18 laboratory collection points, including the main diagnostic laboratory center on the OJHA campus of DUHS in Karachi. Three collection points are in the cities of Sukhur, Moro, and Mirpur Khas, Sindh. Karachi is a metropolitan city in Sindh, Pakistan. Therefore, the information technology department obtained individuals' representative retrospective laboratory data for January, February, and March 2017.

The Roche's Cobas e411 electrochemiluminescence enzyme immunoassay has been used for serum 25(OH)D measurement. It is a fully automated kit with advanced sensitivity and accuracy and is marketed from the California supplier's chain. A serum sample of 10 to 12 µl has been collected from patients in standardized serum collection tubes. The separating gel in a standardized tube keeps 25(OH)D stabilized for 8 h at 20 to 25℃. Centrifugation was carried out in 2 h with a minimum 3.0 ng/ml detection limit of serum 25(OH)D [36].

Over three months, 27,880 individuals were registered in DDRL to assess serum vitamin D levels. Data from symptomatic and asymptomatic male and female individuals of all age groups were obtained from the laboratory to include in the study. Data from individuals with missing age or gender information were excluded from the study. Therefore, the total sample size after exclusion was 26,750 individuals. The independent outcome variable of serum vitamin D levels in males and females of all ages was analysed for vitamin D deficiency, insufficiency, and sufficiency. Individuals with serum vitamin D levels < 20 ng/ml were supposed to consider a vitamin D deficient group, those with values between 21 to 29.9 ng/ml as a vitamin D insufficient group, and those with values > 30 ng/ml as vitamin D sufficient group of population.

The study's primary objective was to estimate the prevalence of vitamin D deficiency, insufficiency, and sufficiency among male and female children, adolescents, adults, and elders.

### Statistical analysis

Statistical analysis was performed using Statistical Package for the Social Sciences software version 22.0. with a 95% confidence interval and a 5% margin of error. Log transformations were applied for the normal distribution of serum vitamin D levels in the study population. Descriptive quantitative analyses were performed for all ages, genders, and demographic variables. Analysis of variance and independent-sample t-test were used to compare mean logs of vitamin D deficiency, insufficiency, and sufficiency among males and females of all ages. *P*-values of < 0.05 were considered statistically significant.

## Results

A total of 26,750 individuals were statistically analyzed for the demographic variables of age and gender correlated with laboratory measurements of serum vitamin D levels.

Table 2 demonstrates descriptive statistics with a mean age of 38 ± 18 of the total population. It was observed that a higher number of females (66%) than males (34%) were included in the sample analyzed for serum vitamin D levels in three months. Vitamin D deficiency was observed in 56% of the total individuals, followed by vitamin D sufficiency in 24% of the individuals and vitamin D insufficiency in 20% of the individuals, respectively.

**Table 2:** General descriptive statistics of demographic variables and serum vitamin D levels

**Baseline Characteristics**	**Total number of individuals** ***n* = 26,750 (%)**	**Serum vitamin D levels ng/ml**	**Mean log serum vitamin D levels ng/ml**^a^
Age in years (mean ± SD)	38 ± 18		
Gender			
Male	9002 (34)		
Female	17,748 (66)		
Vitamin D deficiency	15,031 (56)	< 20	< 1.30
Vitamin D insufficiency	5325 (19)	21 – 29.9	1.30 – 1.48
Vitamin D sufficiency	6394 (24)	> 30	> 1.48

^a^meanlog10data transformed; *SD* Standard deviation; *P*-values were considered significant at 0.05

**Fig. 1:**
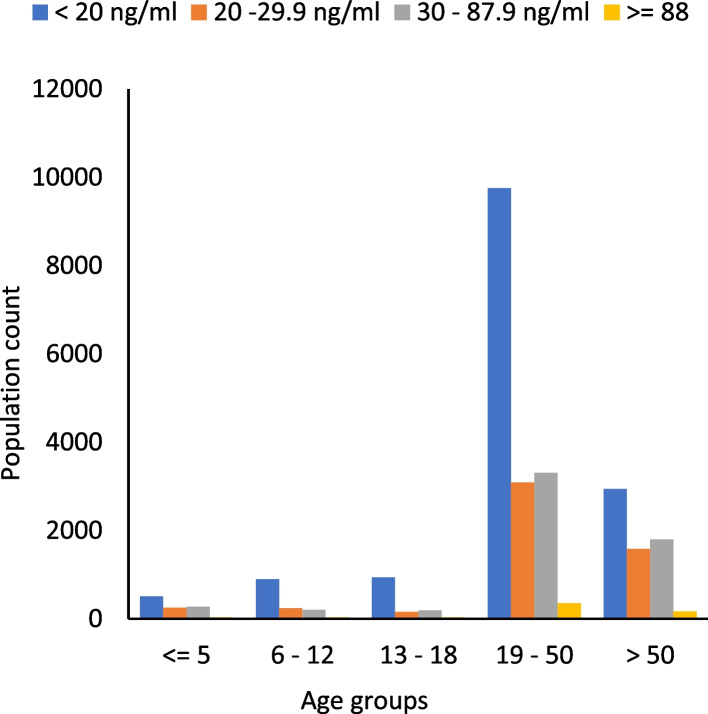
Bar chart comparing vitamin D deficiency, insufficiency, and sufficiency among different age groups

Table 3 and Fig. 1 demonstrate descriptive statistics of the subdivision of the total population into five age groups comprising 4% children with age < 5 years, 5% children aged 6 to 12 years, 5% adolescents aged 13 to 18 years, 62% adults aged 19 to 50 years, and 24% elders aged > 50 years respectively. Table 3 also describes a statistically significant mean logarithmic difference between serum vitamin D levels between these five age groups (*p* < 0.05). However, pairwise comparison using Tukey HSD (α = 0.05) showed no statistically significant mean log difference in serum vitamin D levels of children (≤ 5 years) and elders (> 50 years) with *p*-value > 0.05 so as of children (6 to 12 years) and adults (18 to 50 years) with *p*-value > 0.05. Overall serum vitamin D levels were found to be lower among adolescents (1.12 ± 0.34) followed by children of age 6 to 12 years (1.20 ± 0.30), adults (1.23 ± 0.32), children of age 5 years (1.30 ± 0.32) and elders (1.32 ± 0.30), respectively. Pearson's chi-square statistics for age and serum vitamin D levels revealed a greater vitamin D deficiency in 71% of adolescents, followed by 65% of children aged 6 to 12. In contrast, 24% vitamin D insufficiency was observed in both groups of children aged < 5 years and elders aged > 50 years, respectively. Vitamin sufficiency was found to be higher in elders, followed by children of age < 5 years. There is a statistically significant association between different age groups and vitamin D deficiency, insufficiency, and sufficiency (*p* < 0.05).

**Table 3:** Descriptive statistics of serum vitamin D levels within and between different age groups

Age groups (years)		*N* = 26,750 (%)	Mean log Vitamin D ± SD ng/ml	*P*-Value	Vitamin D deficiency (%)	Vitamin D insufficiency (%)	Vitamin D sufficiency (%)	*P*-Value
Children	< = 5	1067 (3.9)	1.30 ± 0.32	< 0.001*	47	24	26	< 0.001^α^
	6—12	1373 (51)	1.20 ± 0.30		65	18	15	
Adolescents	13—18	1321 (4.9)	1.12 ± 0.34		71	12	15	
Adults	19—50	16,498 (61)	1.23 ± 0.32		59	19	20	
Elders	> 50	6491 (24)	1.32 ± 0.30		45	24	28	

*SD* Standard deviation

^*^One-way ANOVA

^α^Pearson chi-square; *p* values were considered significant at 0.05

**Fig. 2:**
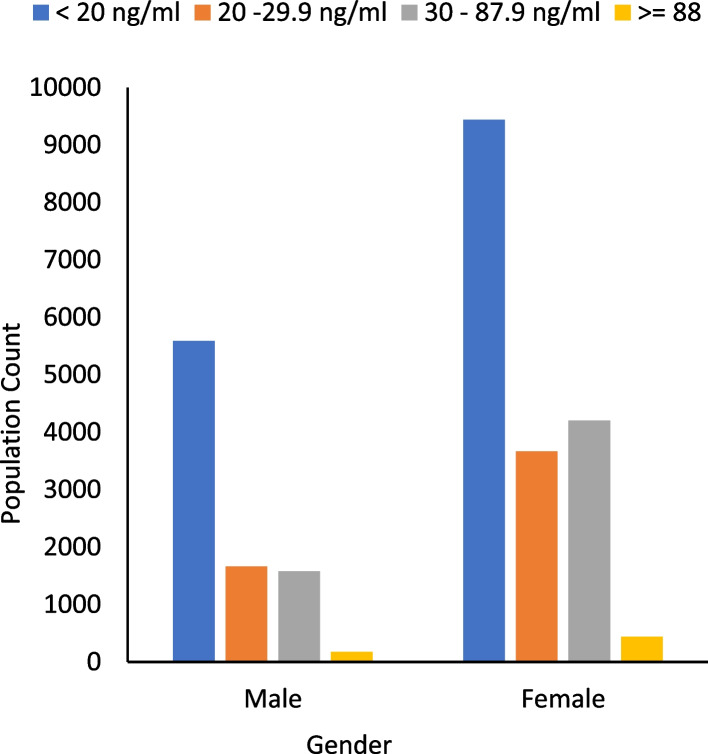
Bar chart comparing vitamin D deficiency, insufficiency, and sufficiency in males and females

Table 4 determines a statistically significant association between gender and serum vitamin D levels in different age groups (*p*-value < 0.05) except for children with < 5 years of age, as shown in Fig. 2. The highest vitamin D deficiency (70%) was observed in females of age ranging from 13 to 18 years, and the highest vitamin D insufficiency (75%) and sufficiency (79%) were observed in females of age ranging from 19 to 50 years, respectively.

**Table 4:** Serum vitamin D levels within and between the different age groups of males and females

Age groups (years)	Gender	*N* = 26,750	Mean log Vitamin D (± SD) ng/ml	Vitamin D Deficiency (%)	Vitamin D Insufficiency (%)	Vitamin D Sufficiency (%)	*p*-value^α^
Overall	Male	9002	1.23 ± 0.29	37	31	27	< 0.001*
	Female	17,748	1.25 ± 0.33	62	69	72	
Children < = 5	Male	625	1.30 ± 0.31	57	59	62	0.621
	Female	442	1.30 ± 0.33	43	41	38	
Children 6—12	Male	762	1.19 ± 0.28	57	58	46	< 0.001*
	Female	611	1.20 ± 0.31	42	42	54	
13—18	Male	432	1.20 ± 0.31	31	37	40	0.004*
	Female	889	1.08 ± 0.35	70	63	60	
19—50	Male	4760	1.20 ± 0.28	33	25	20	< 0.001*
	Female	11,738	1.24 ± 0.33	67	75	79	
> 50	Male	2423	1.28 ± 0.29	44	33	30	< 0.001*
	Female	4068	1.34 ± 0.31	56	66	70	

^α^Pearson chi-square; *p values were considered significant at 0.05. SD: standard deviation

## Discussion

The current study was aimed at the descriptive observations of serum vitamin D deficiency, insufficiency, and sufficiency levels among children, adolescents, adults, and elders of both male and female genders, including both symptomatic and asymptomatic individuals, to observe the total burden of vitamin D deficiency and insufficiency in Pakistan. Previously published scientific literature documented the high prevalence of serum vitamin D deficiency in American (25%), Canadian (36%), European (40%), and South Asian populations (61%), addressing different age groups [39, 41–43]. To the best of our knowledge, the current study included the largest cohort of individuals of age ranging from birth to more than fifty years and both male and female genders from rural and urban Pakistani populations. The female-to-male ratio was greater (2:1) in the statistically analysed population.

Serum vitamin D deficiency was found to be 56% of the overall population. It has been observed that it was higher in females than in males. Regarding serum vitamin D insufficiency, about 20% of the general population was affected, with a higher female-to-male ratio. Therefore, it can be stated that the prevalence of vitamin D deficiency and insufficiency is greater in females than in males. These findings can be attributed to a larger cohort of females than males. In previous literature, Hussain et al. reported vitamin D deficiency in 59% of female housewives in Quetta, Pakistan. Factors attributed to a higher prevalence of vitamin D deficiency in women were found to be lack of education, sunlight exposure of fewer than 15 min on weekdays, the behavior of avoiding sun exposure or keeping themselves covered in sunlight, less intake of eggs, milk, or fish for only one or two times a week or no intake at all [44]. These findings can also be correlated with the findings of other studies conducted in Punjab and Khyber Pakhtunkhwa, demonstrating 54% deficiency and 31% insufficiency of serum vitamin D levels among asymptomatic individuals [45].

For the child population, male children aged 12 years appeared to have greater vitamin D deficiency of 57% and insufficiency of 60% than female children. This higher percentage of vitamin D deficiency in male children can be attributed to a higher number of males than females in the cohort of children. Serum vitamin D levels of children aged 6 to12 years and those of adolescents were lower than adults, despite smaller cohorts compared to adults. Nutritional deficiencies in growing children, such as iron deficiency in anemia associated with vitamin D deficiency, were found common in a previous study conducted in Pakistan [46]; hence, it can be attributed as a causative factor.

Diet is an essential factor in meeting the body's vitamin D requirements. It has been observed by Crowe et al. that meat and fish eaters have better serum vitamin D levels compared to vegetarians and vegans [47]. Through self-management, fortified milk and dairy products help to overcome vitamin D deficiency [48].

Ultraviolet sun rays penetrate the skin and are essential in synthesizing vitamin D [8]. The lower solar zenith angle facilitates the effective absorption of ultraviolet B (UVB) radiation for vitamin D synthesis [49]. It has been observed that the ozone layer absorbed 99% of UVB radiation, and only 1% reached the earth at a higher solar elevation angle. The city of Karachi lies at 24^O^N /67 ^O^E on the global map, and the solar elevation angle is maximum between 10:00 and 15:00 h for maximum absorption of UVB for vitamin D synthesis [48].

The average annual temperature in Karachi lies between 20 and 31^O^C. The average monthly sunshine hours are 125 to 300 [50]. An observational study revealed that sunlight for 10 to 15 min. twice a week is sufficient to make serum vitamin D3 in the body [8]. The published literature reported the exposure of individuals to sunlight for more than 30 min. a week with two to three exposed body surfaces, such as faces, arms, or hands, in Karachi [51]. The endemic status of vitamin D deficiency and insufficiency was found alarming despite the good exposure of the Pakistani population to sunlight.

A survey was conducted at Agha Khan University, Karachi, in 2015 and reported the prevalence of vitamin D deficiency up to 61% in ambulatory individuals coming to general OPD [17]. These findings are closer to the results of the current study. Previous literature revealed that vitamin D deficiency could be associated with decreased serum calcium levels and elevated levels of serum phosphate and alkaline phosphatase [32]. The higher frequency of vitamin D deficiency could be due to indoor lifestyles, a lack of awareness and understanding of the time needed for vitamin D in the sunshine, and meals enriched with vitamin D [52]. Hence, the study results indicate that the public and private health sectors must implement preventive and treatment measures and public awareness programs to control the rapidly spreading condition of vitamin D deficiency and insufficiency.

The limitations of this study can be stated as the contributing factors of vitamin D deficiency and insufficiency related to any associated disease, the nutritional behavior of study participants, and primary data regarding their exposure time to sunlight could not be evaluated. The participants included in the study were patients referred from general OPD for laboratory testing of serum vitamin D levels because their doctor suspected low vitamin D levels, which could have inflated the reported prevalence of vitamin D deficiency and insufficiency of the sample. Moreover, Due to procedural constraints of data extraction from the laboratory and time limitations, the study was carried out for a dataset of three months, from January to March 2017.

This study could have some selection bias, as only the vitamin D levels of individuals prescribed for laboratory tests were reported, as opposed to screening non-symptomatic individuals for vitamin D levels.

The strength of this study can be attributed to the best possible estimation of serum vitamin D deficiency and insufficiency status among children, adolescents, adults, and elders in the developing country of Pakistan, with a large sample size to generalize the study results to the study population. Because children and adolescents appear to be the most affected age group, it is critical to implement awareness campaigns about causative factors, preventive measures, and treatment modalities to manage serum vitamin D levels.

## Conclusions

The findings of vitamin D deficiency in children and adolescents direct higher authorities in the public and private health sectors to take immediate steps for screening and educating the high-risk population and intervene in the affected population with vitamin D supplements to establish preventive and therapeutic measures.

## Data Availability

The secondary dataset was used for the current study and is available at https://doi.org/10.6084/m9.figshare.20003174.v1.

## Abbreviations

OPD: Out-Patient-Department
DDRRL: Dow Diagnostic Research and Reference Laboratory
DUHS: Dow University of Health Sciences
STROBE: Strengthening the Reporting of Observational Studies in Epidemiology
25(OH)D: 25 Hydroxyvitamin D; UVB: Ultraviolet B
